# Gold nanoparticles-loaded anti-miR221 enhances antitumor effect of sorafenib in hepatocellular carcinoma cells

**DOI:** 10.7150/ijms.37427

**Published:** 2019-10-21

**Authors:** Hongqiao Cai, Yang Yang, Fenghui Peng, Yahui Liu, Xueqi Fu, Bai Ji

**Affiliations:** 1 Department of Hepatobiliary and Pancreatic Surgery, the First Hospital, Jilin University, Jilin 130021, China;; 2 Edmond H. Fischer Signal Transduction Laboratory, School of Life Sciences, Jilin University, Changchun, Jilin 130012, China.

**Keywords:** hepatocellular carcinoma, sorafenib, gold nanoparticles, miR-221, signaling pathway

## Abstract

**Objective:** Currently, sorafenib is the main systemic chemotherapy drug for advanced stage of hepatocellular carcinoma (HCC). However, emerging data from some clinical HCC patients indicates that sorafenib alone has only moderate antitumor efficacy, and could not inhibit metastasis and progression of disease. MiR-221 plays a role in promoting tumorigenesis in HCC by inhibiting the expression of p27. In this study, we analyzed the synergistic anti-tumor effects of sorafenib and gold nanoparticles-loaded anti-miR221 on HCC cell lines.

**Methods:** Gold nanoparticles-loaded anti-miR221 was investigated and identified by transmission electron microscope, ultraviolet-visible spectroscopy, zeta potential and dynamic light scattering measurements as well as the confocal microscopy and dark-field imaging. Two HCC cell lines were treated with sorafenib and AuNPs-anti-miR221 alone or combination *in vitro* to investigate the inhibitory effect by CCK-8, live/dead fluorescence staining and colony-forming unit assays. MiR-221/p27/DNMT1 signaling pathway including p27 and DNMT1 was examined by western blot.

**Results:** AuNPs-anti-miR221 can enhance the effect of sorafenib in inhibiting cell proliferation via inactivating miR-221/p27/DNMT1 signaling pathway.

**Conclusions:** Our results demonstrate that sorafenib combined with AuNPs-anti-miR221 treatment does effectively inhibit proliferation of HCC cell lines synergistically. These data suggest the AuNPs-anti-miR221 may be a promising chemosensitizer to sorafenib in the treatment of HCC.

## Introduction

Hepatocellular carcinoma (HCC) is the third leading cause of cancer mortality worldwide, with an increasing incidence in the United States and China 1, 2. In China, HCC commonly arises in patients with chronic liver diseases. Only HCC patients in early stage are amenable to potentially curative therapies, including surgical resection and liver transplantation. In the present, the multi-kinase inhibitor sorafenib is the main systemic chemotherapy medicine to improve survival in those patients with advanced HCC 3. However, some patients only show moderate or mild response to sorafenib. Therefore, prognosis of advanced HCC remains poor, and new effective therapeutic strategies are urgently needed. To find efficient targets, a number of large-scale molecular studies have been conducted in HCC, including miR-221^4^. MiR-221 is a non-coding microRNA and can promote HCC malignancy by inhibiting the expression of p27. Therefore, miR-221 plays an important role in HCC proliferation and metastases 5.

Nanoparticles have emerged as new carriers for anti-cancer drugs. Gold nanoparticles (AuNPs) are thought to be suitable drug carriers in tumor diagnosis and treatment due to small size, great biocompatibility and precise targeting ability 4, 6. Based on the unique structure of AuNPs, researchers make use of their high surface area to amount ratio. Also, external functionalization of AuNPs extends their biomedical application dramatically. A number of functional groups including peptides, oligonucleotides and antibodies could be modified onto the surface of AuNPs 7.

The miR-221/p27/DNMT1 signaling pathway is a promising target with respect to its frequent dysregulation in HCC and its key role in regulating cell proliferation, migration, survival and angiogenesis. Aberrant miR221 signaling has been detected in nearly half of hepatocellular carcinoma, and a correlation between poor outcome and miR221 signaling activation has been shown in patients with treatment of sorafenib^8^-^10^.

**Scheme 1:**
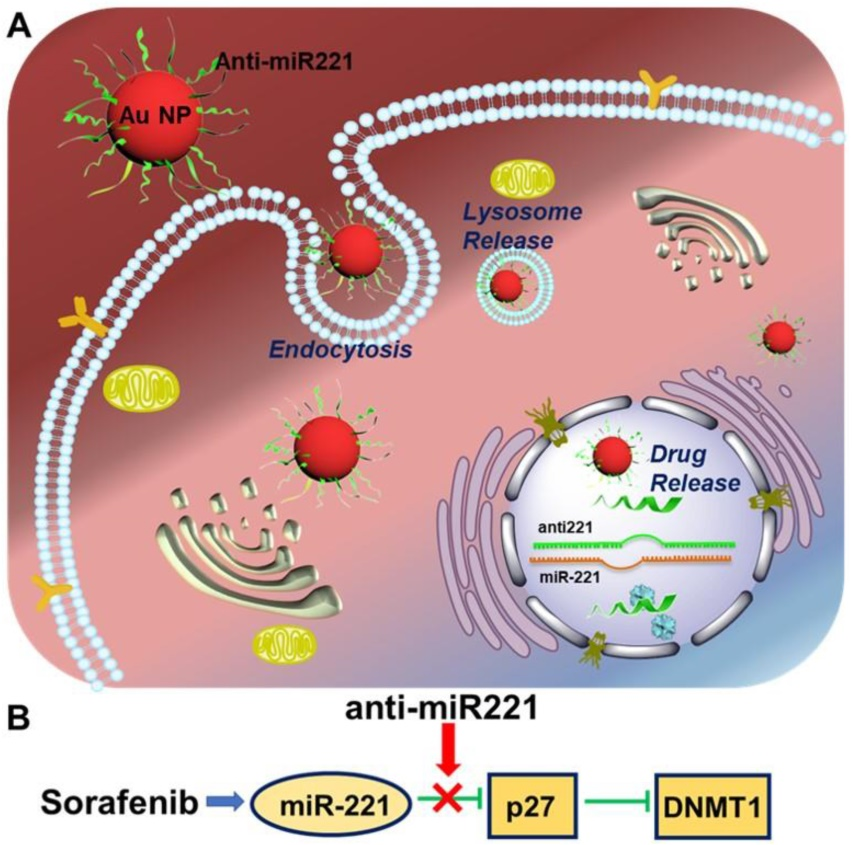
Illustration of AuNPs-anti-miR221 target miR221/P27/DNMT1 pathway in sorafenib-treated HCC cells

Currently, sorafenib plays a critical role in treating patients with advanced HCC, contributing to an improved overall survival of treated patients in clinical trials 11. Unfortunately, some patients couldn't acquire the anticipated treatment effect 12-15. It is imperative to investigate the potential molecular mechanism resulting in the low survival benefits, in order to develop potential strategies aimed at increasing its efficacy against HCC. Hence, this study is to investigate the antitumor effect of AuNPs-anti-miR221 in order to overcome the resistance of sorafenib in HCC treatment. In this study, we show that gold nanoparticles-loaded miR221 inhibitor can enhance the effect of sorafenib through downregulation of p27 and upregulation of DNMT1. In addition, they present a synergistic effect in inhibiting cell proliferation. These results suggest that anti-miR221 may be a novel chemosensitizer to increase chemotherapeutic sensitivity of sorafenib on HCC cells (Scheme 1).

## Materials and Methods

### Chemicals and antibodies

Sorafenib (Santa Cruz Co.) was dissolved in DMSO to prepare the stock solution of 20mM and stored in aliquots at -20°C. Antibodies against P27, DNMT1 and β-actin were purchased from cell signaling Technology.

### Cell lines and culture conditions

Hepatocellular carcinoma cell lines, HepG2 and Huh7 purchased from ATCC were cultured in DMEM supplemented (Hyclone, Logan, UT, USA) with 10% FBS ( Hyclone, Logan, UT, USA) and 1% of penicillin-streptomycin at 37℃,in humidified air containing 5% CO_2_. HepG2 cells generated from a hepatoblastoma that resected from a young Argentinian patient were used as a model of hepatoma cells.

### Preparation of AuNPs- anti-miR221

The small size gold nanoparticle was firstly synthesized via a classic method reported by Turkevich 16. 1 mL of 1.0 % HAuCl4 was added in the 98 mL of deionized water and heated this mixture solution with stirring until it closed to boiling. Then 1 mL of 1.0% trisodium citrate was added in it and continually heated it for around 30 min. During this process, the color of the solution changed from colorless to red wine. After 30 min, stop the heat and keep stirring before it cooled to room temperature. The synthetic gold nanoparticles were chartered by UV-Vis spectroscopy, TEM and DLS, respectively.

Purchased anti-miR221 (SH-TTTTTTTTTTTGAAACCCAGCAGACAATGTAGCT) was dissolved in the RNase-free buffer with a concentration of 50 μM. 15 mM tris(2-carboxyethyl) phosphine (TCEP) was added in this solution for a short time to break the disulfide of the anti-miR221. To obtain anti-221 coated AuNPs, 10 mL of AuNPs was firstly co-cultured with 1.0% SDS with stirring at room temperature for 24 h, and then 50 μL of the 50 μM anti-miR221 was added in the SDS stabled AuNPs solution with the ration of 200:1. After 1 h later, 300 μL of 2.0 M NaCl buffer (0.01% SDS, 0.01 M PB) was added in this solution and sonicated it for 1 min to improve the DNA loading. Repeat this step three times within 24 hours. To remove excess anti-miR221, the mixture solution was centrifuged and the AuNPs-anti-miR221 precipitate was resuspended in PBS buffer for the further use.

### Cell proliferation assay

Cell proliferation assays were performed using Cell Counting Kit-8 (CCK8, Dojindo Molecular Technologies) following manufacturer's instructions. Briefly, cells were seeded into 96-well microplates and nanoparticles and sorafenib alone or combination were added after 24h of incubation. The cells were cultured for another 48h, and 10 μL of CCK8 was added to each well. The microplates were incubated at 37^o^C for 0.5 h. Absorbance was read at 450 nm using a microplate reader. At least three independent experiments were collected.

### Colony-forming assays

Colony-forming assays were performed using Wright-Giemsa stain (Baso, CHN) following manufacturer's instructions. Briefly, cells were seeded into 24-well microplates and sorafenib was added after 24h of incubation. The cells were cultured for another 48h, and 300μl Wright-Giemsa stain was added to each well. The microplates were incubated at 37°C for 1 min. Then, we rinsed it with double-distilled water gently for three times, dried and examined the finished slide under a microscope. Finally, the 24-well microplates were photographed by using scanner (Epson Perfection V370 Photo).

### Calcein-AM/PI staining

Fluorescence stain were performed using Calcein-AM/PI (Dojindo Molecular Technologies) following manufacturer's instructions. Briefly, cells were seeded into 24-well microplates and sorafenib was added after 24 h of incubation. Cells were incubated for another 48h. The culture media was removed and the cells were washed with PBS for three times. After that, 300 μL of prepared Calcein-AM/PI was added to each well. The microplates were incubated at 37 °C for 20 min. After incubation, the cells were washed with PBS for two times, and examined by fluorescent microscopy. The living cells were stained green and the dead cells were dyed red.

### Western blotting

The procedures were followed as the standard protocol 17. After various treatments, the whole cellular lysates were prepared by harvesting the cells in 1X cell lysis buffer [20 mM HEPES (pH 7.6), 150 mM NaCl and 0.1% NP40] supplemented with 1X phosphatase inhibitor Cocktail 2 and 3 (Sigma-Aldrich), 1 mM PMSF (Sigma-Aldrich) and 1X protease inhibitors (protease inhibitor cocktail set III, Calbiochem-Novabiochem, San Diego, CA, UA). Protein was resolved by sodium dodecyl sulfate (SDS)-polyacrylamide gel electrophoresis and transferred onto PVDF membranes (Amersham, Piscataway, NJ, USA). The antibodies used were β-actin (Santa Cruz Biotechnology); DNMT1 (Santa Cruz Biotechnology), P27 (Cell Signaling Technology). Quantification of the bands was analyzed using software Image J.

### RNA isolation, cDNA preparation and quantitative PCR (qPCR)

The protocol was followed as previously reported 18. The miRNeasy Kit (Qiagen) and High Capacity cDNA Reverse Transcription Kits (Applied Biosystems) was used to isolate RNA and reverse transcription for cDNA respectively. To measure the miR221 expression, Taqman technology (Applied Biosystems) was carried out. For normalization, the ΔCT approach was used to analyze the expression of the 18 S levels (Forward: ACAGGATTGACAGATTGA; Reverse: TATCGGAATTAACCAGACA).

### Statistical analysis

All data were presented as mean ± SD. Student's t-Test was used for comparison between two groups. One-way ANOVA was used to compare difference of multiple groups. Synergistic effect between sorafenib and AuNPs-anti-miR221 in HepG2 and Huh7 cells are calculated using Chou-Talalay method. P < 0.05 was considered statistically significant.

## Results

### Sorafenib inhibits HCC cell lines proliferation

**Figure 1:**
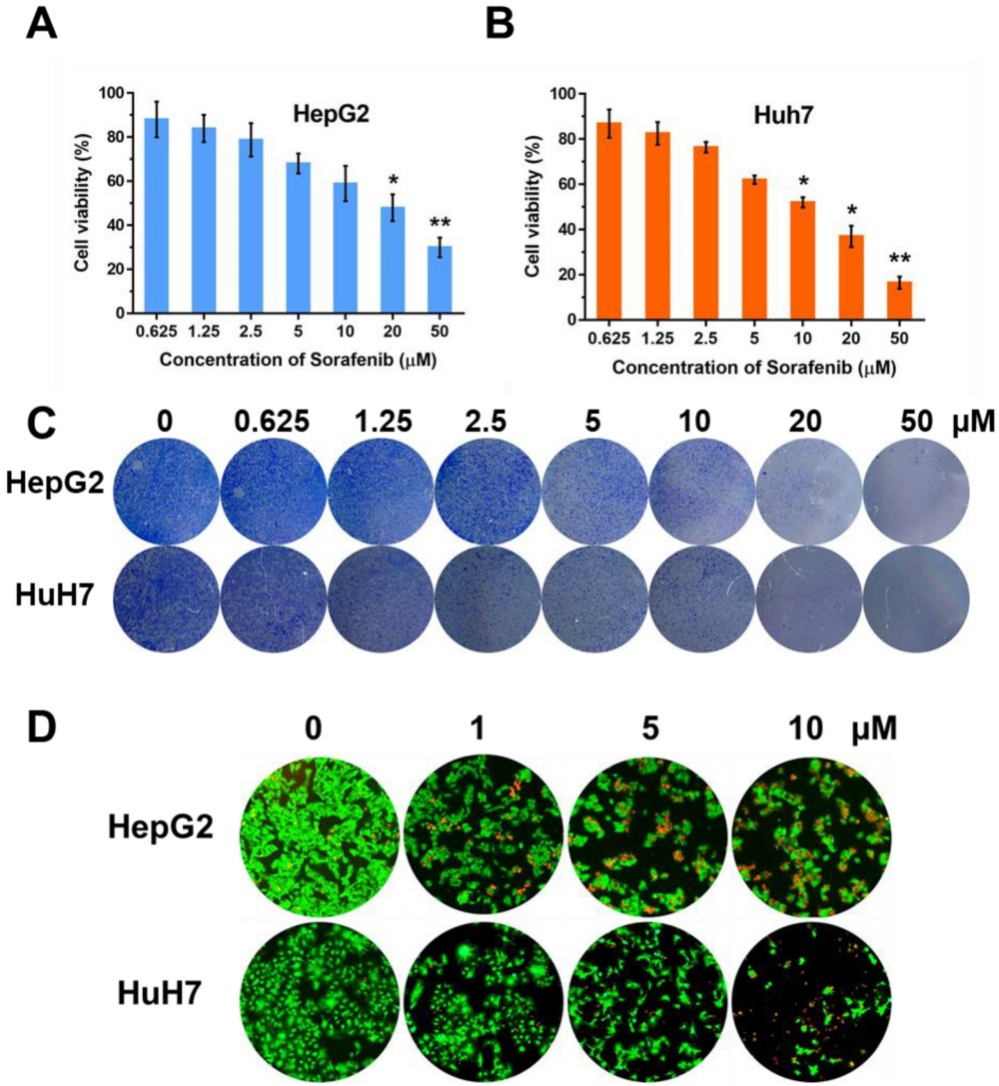
** The effect of sorafenib on cell proliferation**. Cell viabilities of HepG2 (A) and Huh7 (B) after treatment of sorafenib; (C) Colony formation of HepG2 and Huh7 cells; (D) Fluorescence stain of living and dead cells. The experiments are repeated for three times. Data are mean ± SD; *P < 0.05, **P< 0.01.

In order to observe the inhibition effect of sorafenib in cell proliferation, we used CCK-8 approach to test the cell viabilities on two HCC cell lines, HepG2 and Huh7, with the treatment of sorafenib (0, 0.625, 1.25, 2.5, 5, 10, 20 µM). Moreover, colony formation was conducted and the concentration of sorafenib used was the same as that in CCK-8 approach. The results of CCK-8 (Figure 1A, B) and colony formation (Figure 1C) suggested that sorafenib led to a dose-dependent inhibition on cell proliferation. Furthermore, we selected three dosages (1, 5, 10 µM) of sorafenib to carry out the fluorescence staining of living and dead cells, the result of which was consistent with the former experiments (Figure 1D).

### Sorafenib activated miR-221/p27/DNMT1 signaling pathway

**Figure 2:**
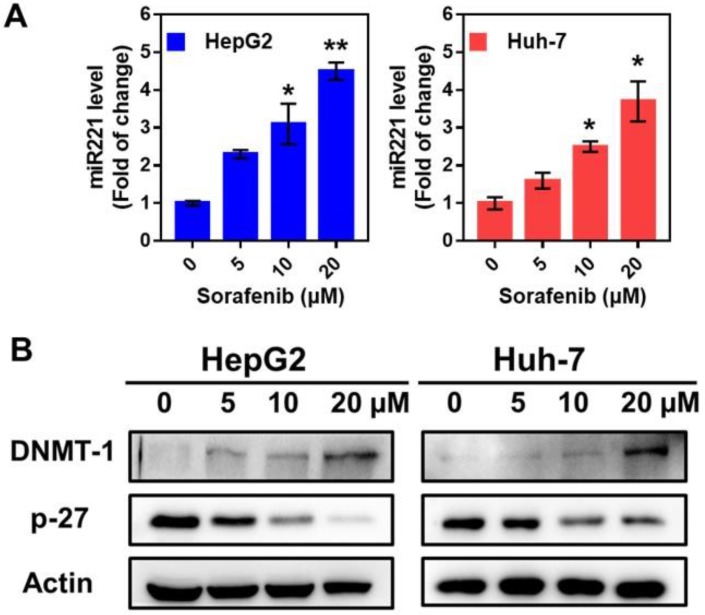
** The impact of sorafenib on miR221 expression and miR-221/p27/DNMT1 signaling pathway.** (A) miR221 level after treatment of sorafenib; (B) Western blot of the DNMT1, p27 and β-actin expression. Graphs indicate the miR221 level from 3 independent experiments. Data are mean ± SD; *P < 0.05, **P< 0.01.

Sorafenib treatment in HepG2 and Huh7 cells activated miR221 signaling pathway, which led to miR221 overexpression. The qPCR was used to measure the expression of miR221 in mRNA level. After the treatment of sorafenib (with different concentration of 0, 5, 10, 20 µM), the miRNA level was significantly increased in HepG2 and Huh7 cells (Figure 2A). As shown in Figure 2B, the western blot confirmed the downregulation of sorafenib (with different concentration of 0, 5, 10, 20 µM) on p27, and the consequent upregulation on DNMT1.

### Synthesis and identification of AuNPs-anti-miR221

**Figure 3:**
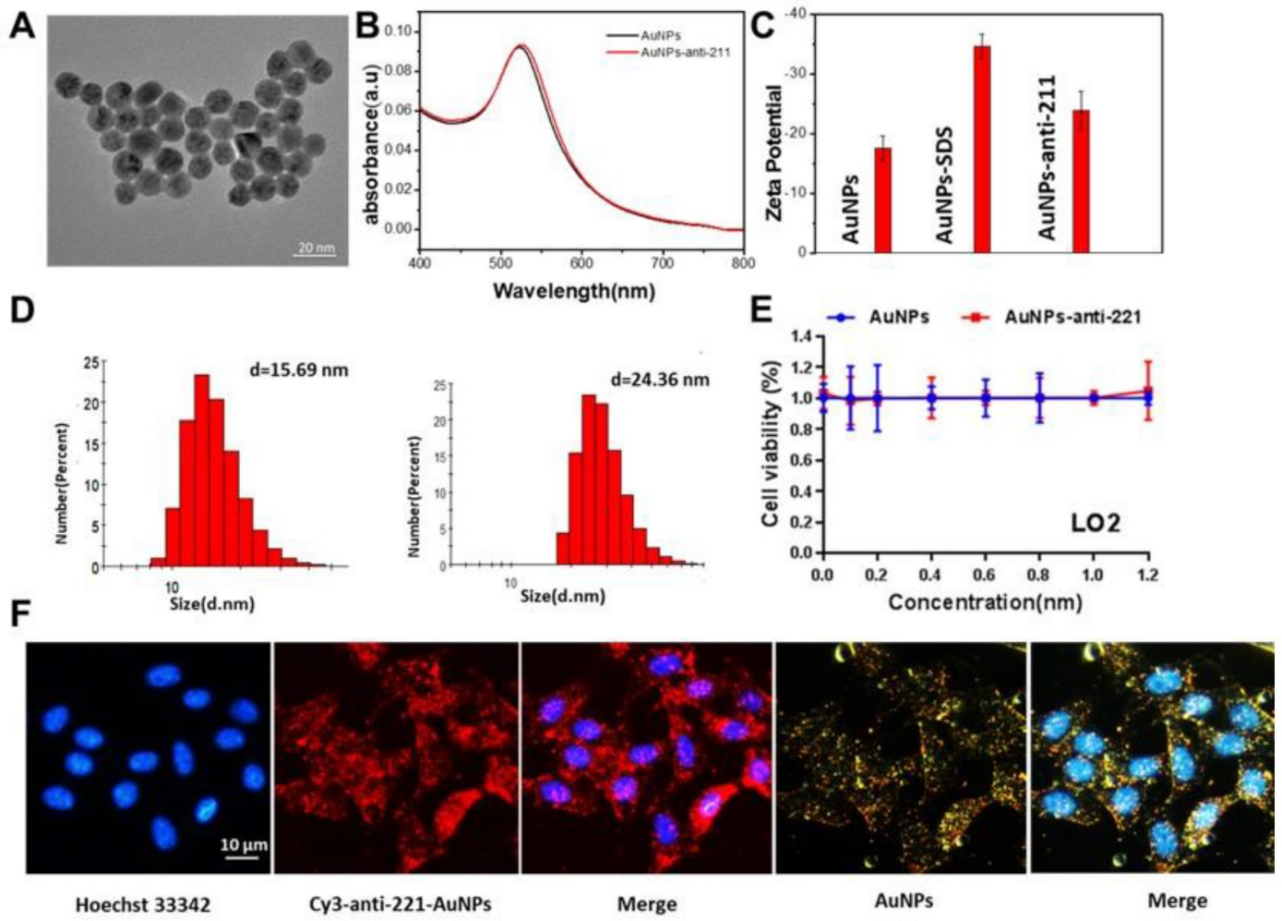
***In vitro* characterization of AuNPs-anti-miR221.** (A) TEM images; (B) Absorption bands; (C) Zeta potential; (D) Dynamic light scattering measurements; (E) The viability of LO2 after treatment of AuNPs or AuNPs-anti-miR221; (F) Fluorescence and dark-field imaging showing the nuclear targeting by AuNPs-anti-miR221. Blue, red and yellow represent the nuclei stained by Hoechst 33342, Cy3 labeled anti-miR221 and dark-field of AuNPs. All experiments were repeated three times and values are presented as mean ± SD. Note: AuNPs-anti-221 and Cy3-anti-221-AuNPs abbreviations indicate AuNPs-anti-miR221 and Cy3 labeled AuNPs-anti-miR221 respectively.

To examine the functional nanoparticles, we performed a series of characterization assays. From the image taken by transmission electron microscope (TEM), we found that the diameter of AuNPs was about 13 nm (Figure 3A). The ultraviolet-visible spectroscopy showed nearly no change in absorbance after modification of anti-miR221 on AuNPs (Figure 3B). Zeta potential and dynamic light scattering measurements were further conducted and confirmed successful modification of anti-miR221 on AuNPs (Figure 3C, 3D). Next, we evaluated the cytotoxicity on normal hepatocellular cells. We treated LO2 cells with different concentrations of AuNPs or AuNPs-anti-miR221 (0, 0.2, 0.4, 0.6, 0.8, 1, 1.2 nmol/L). The result of CCK showed that AuNPs and AuNPs-anti-miR221 had little cytotoxicity (Figure 3E).

To evaluate the cellular uptake of functionalized nanoparticles towards hepatocellular carcinoma cells, the confocal microscopy assay and dark-field imaging were used. As shown in Figure 3F, the nucleus of HepG2 cells were dyed with blue florescence, and the cy3 labeled AuNPs-anti-miR221 displayed both red and yellow (dark-field) florescence. The strong florescence signal indicated that AuNPs-anti-miR221 had improved targeting ability towards hepatocellular carcinoma cells.

### AuNPs-anti-miR221 and sorafenib inhibits hepatocellular carcinoma cell lines proliferation synergistically

**Figure 4:**
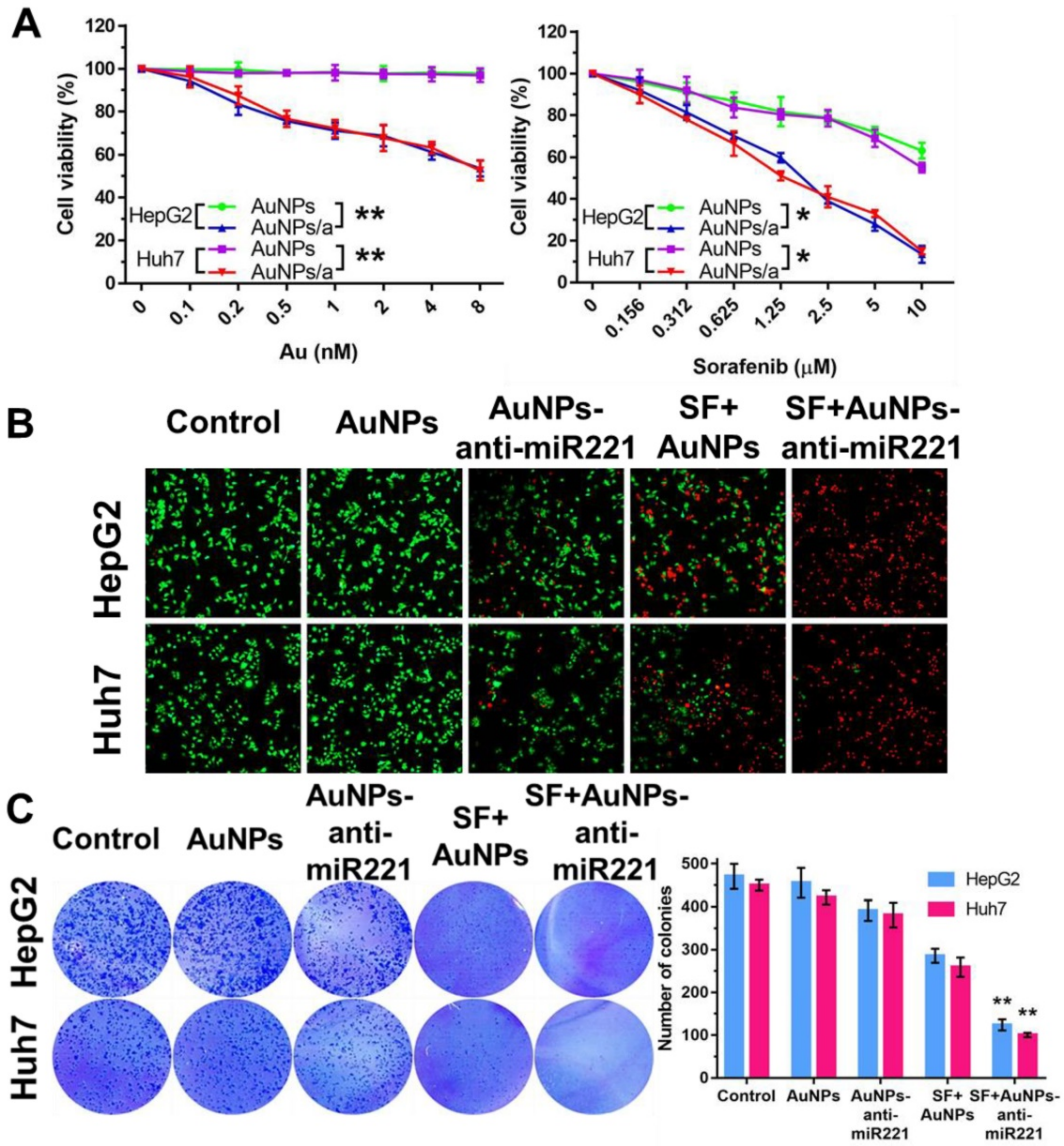
** Combination treatment of AuNPs-anti-miR221 and sorafenib**. (A) Cell viabilities in HepG2 and Huh7 using CCK-8 assay; (B) Fluorescence staining of HepG2 and Huh7 cells; (C) Colony forming assays. Graphs indicate the colony number from 3 independent experiments. Data are mean ± SD; *P < 0.05, **P< 0.01. Note: AuNPs/a abbreviations indicates AuNPs-anti-miR221.

In order to examine synergistic effect of AuNPs-anti-miR221 and sorafenib *in vitro*, we investigated the effects of two drugs on two different HCC cell lines using CCK-8 assay, colony formation and live/death fluorescence staining. As shown in Figure 4A, the AuNPs showed nearly no impact on cell viability, while AuNPs-anti-miR221 with increasing concentration (0, 0.1, 0.2, 0.5, 1, 2, 4, 8 nmol/L) could inhibit cell growth in both HepG2 and Huh7 cells. When AuNPs-anti-miR221 (0.1 nmol/L) was incubated with sorafenib (0, 0.156, 0.312, 0.625, 1.25, 2.5, 5, 10 µmol/L), the inhibition of cell growth was dramatically enhanced. To study the synergistic effect, the combination index was further calculated using Chou-Talalay method, and the result displayed a high synergistic effect between sorafenib and AuNPs-anti-miR221 in both HepG2 and Huh7 cells. The HepG2 and Huh7 cells were grown in 24-well plate and were exposed to AuNPs-anti-miR221 (5 nmol/L), sorafenib (5 µmol/L) and combination respectively. After 48h, cell viability was examined. As shown in Figure 4B, 4C, AuNPs-anti-miR221 can enhance sorafenib inhibition effect in a synergistic manner.

### Combination of AuNPs-anti-miR221 and sorafenib inhibits miR221/P27/DNMT1 pathway activation

**Figure 5:**
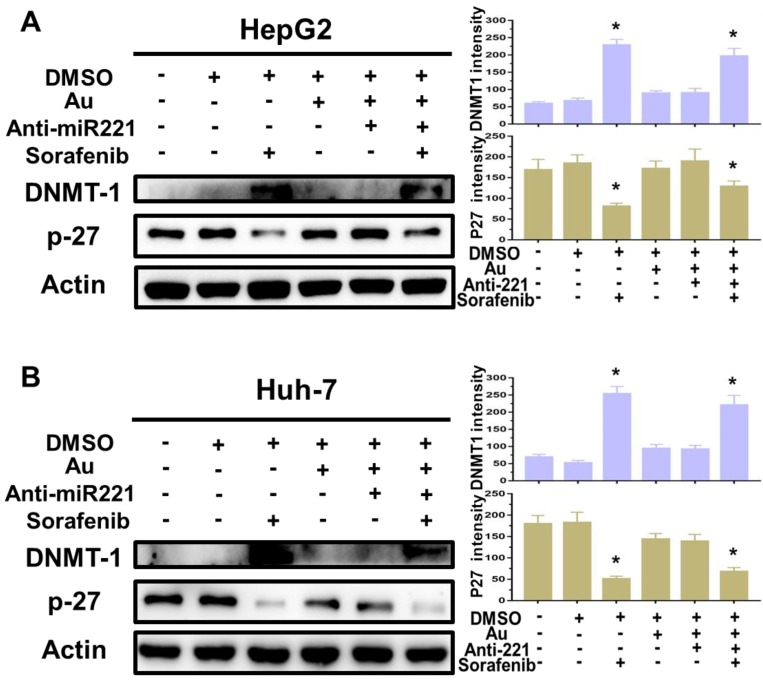
** The impact of AuNPs-anti-miR221 and sorafenib treatment on miR221/p27/DNMT1 signaling pathway**. Western blot of the DNMT1, p27 and β-actin expression as well as the quantification of bands in HepG2 (A) and Huh7 cells (B). Data are mean ± SD; *P < 0.05.

Finally, to better understanding the molecular mechanisms of synergistic effect of sorafenib and AuNPs-anti-miR221 in HCC cells, we incubated two HCC cell lines with AuNPs-anti-miR221 (5 nmol/L) or sorafenib (5 µmol/L) alone or combination for 24 h, and the levels of p27 and DNMT1, downstream targets of miR221, were detected by western blot. As shown in figure 5A and 5B, treatment with sorafenib and AuNPs-anti-miR221 combination results in increased expression of p27 and decreased expression of DNMT1, which demonstrates that AuNPs-anti-miR221 can work as a chemosensitizer to sorafenib.

## Discussion

Sorafenib is the main systemic chemotherapeutic drug for HCC patients in advanced stage. Our results indicated the conspicuous effect of sorafenib on inhibiting hepatocellular carcinoma cells. However, both clinical cases and recent studies have revealed that it is not fully effective in preventing recurrence and progression because of low response or resistance, which has become a barrier to further improve the survival time and benefit the HCC patients 19. Many research groups focus on the molecular mechanisms in sorafenib resistance in search of the established therapeutic agents that can overcome the resistance in HCC, and help develop potential strategies aimed at increasing its efficacy against HCC 20. The pre-existence and rapid acquirement of powerful mechanisms of chemoresistance (MOC) may account for the low efficacy of sorafenib in HCC, and a reduction in intracellular concentrations is found to play an important role among different types of MOC involved in multidrug resistance (MDR) phenotype 21,22. The sensitization of HCC to sorafenib could be enhanced either by ABC-mediated efflux or OCT1-mediated uptake to increase the intracellular content of this drug 21,22. Besides the intracellular drug concentrations, the response of cells to drug is another factor affecting resistance. Since microRNAs affect direct and indirect drug response through the regulation of important genes expression 23, it is tempting to hypothesize that a mechanism may be correlated between miR221 and sorafenib resistance in HCC.

P27 is an important transcriptional regulator, the expression of which inhibits cell apoptosis and promoted cell proliferation 24, 25. DNA methyltransferase 1 (DNMT1) is the primary enzyme that maintains DNA methylation during replication, whose dysregulation could result in a variety of diseases 26, 27. From the perspective of miR-221/ p27/DNMT1 signaling pathway, the decreased expression of p27 and increased expression of DNMT1 resulted in the decrease of cell growth. To partially reverse the overexpression of miR221, we used anti-miR221 to specifically bind with miR221 in the cell nucleus. However, considering the degradation of the synthetic nucleotide like miRNAs, a drug carrier is urgently needed. Nanoparticles can improve the drug stability by decreasing degradation, and achieve the goal of active targeting through surficial modification. Therefore, given the regulation of miR221/p27/DNMT1 signaling pathway, we designed the gold nanoparticles (AuNPs) loaded with anti-miR221, a specific inhibitor of miR221, to activate the expression of p27 and suppress the expression of DNMT1, which could finally enhance the effect of sorafenib in HCC.

The size of AuNPs-anti-miR221 is small enough to take the enhanced permeability and retention (EPR) effect for solid tumor 28. Small size makes it easier to overcome the barrier of vascular wall and get to the target. The blood vessel is known to have negatively charged surface, so that particles with negative charge such as AuNPs-anti-miR221 will decrease the nonspecific binding and rapid clearance. Little cytotoxicity endows AuNPs-anti-miR221 good biocompatibility for further application in anti-cancer drug delivery because of the little impact on normal cell and tissues. The uptake of AuNPs is consist of its adsorption onto cell membrane and internalization into cell 29. The citrate-stabilized AuNPs may contain different kinds of proteins on its surface, which are involved in the passive uptake of AuNPs mediated by nonspecific adsorption of serum proteins and receptor-mediated endocytosis pathway 30,31. The modification of anti-miR221 even may increase the cellular uptake, which possibly contributes to the specific binding of miR-221.

Some researchers found that miR221 plays a critical role in sorafenib resistance through inhibition of Caspase-3-Mediated apoptosis 32. In our study, we found that sorafenib could activate miR221 and promote cell proliferation. Therefore, it is a significant target in search for drugs that can be used as chemotherapeutic agents for cancer. Anti-miR221 can inhibit cell proliferation and induce apoptosis, but there is no report regarding its effect on HCC cells. In this study, we showed that AuNPs-anti-miR221 alone treatment had only a minor effect on proliferation of HCC cell lines, and sorafenib alone treatment had a moderate inhibition effect. However, combined sorafenib with AuNPs-anti-miR221 treatment led to a strong synergistic effect on proliferation of HCC cells.

These data demonstrate that sorafenib and AuNPs-anti-miR221 combination treatment exerts anti-cancer effect on HCC via inhibiting miR221/p27/DNMT1 signaling pathway. Therefore, we hypothesize that AuNPs-anti-miR221 may be a chemosensitizer to sorafenib for advanced HCC patients. This study has therefore provided a framework for the development of sorafenib-based combination therapies for HCC.

## Conclusion

In conclusion, the results of this study demonstrate that combining sorafenib and AuNPs-anti-miR221 shows a synergistic effect in HCC cell lines. Therefore, this combination treatment strategy may be a promising treatment option for patients with advanced HCC.
